# Alignment and/or ligament balancing? Towards truly personalized total knee arthroplasty—A narrative review

**DOI:** 10.1002/jeo2.70713

**Published:** 2026-04-09

**Authors:** Grégoire Micicoi, Peter K. Sculco, Stefano Zaffagnini, Friedrich Boettner, Michael T. Hirschmann, Sébastien Lustig

**Affiliations:** ^1^ IULS‐University Institute for Locomotion and Sports, Pasteur 2 Hospital University Côte d'Azur Nice France; ^2^ Complex Joint Reconstruction Center, Hospital for Special Surgery New York New York USA; ^3^ 2nd Orthopedics and Trauma Unit IRCCS Istituto Ortopedico Rizzoli Bologna Italy; ^4^ University Department of Orthopaedic Surgery and Traumatology Kantonsspital Baselland Bruderholz Switzerland; ^5^ Department of Orthopaedics, Croix Rousse Hospital Claude Bernard Lyon 1 University Lyon France

**Keywords:** alignment philosophy, knee laxity, ligament balancing, personalized surgery, phenotypes, total knee arthroplasty

## Abstract

Total knee arthroplasty has evolved from a purely mechanical procedure towards a more personalized restoration of each patient's native knee function. This narrative review explores the physiological and pathological patterns of knee laxity, the spectrum of alignment phenotypes and their interplay with ligament balancing strategies. It summarizes current evidence on physiological laxity envelopes, osteoarthritic alterations and alignment classifications such as Coronal Plane Alignment of the Knee, functional and laxity‐based phenotypes. The review further examines how alignment philosophies affect soft‐tissue tension and postoperative stability. Recent advances in robotic and sensor‐assisted techniques have enabled objective intraoperative assessment of ligament balance, allowing reproducible quantification of medial–lateral gaps and compartmental pressures. These technologies reveal that individualized ligament behaviour often diverges from bony alignment, underscoring the need to define reproducible physiological balance targets. Functional outcomes appear optimized when medial stability and controlled lateral laxity are restored, supporting the concept of reproducing physiological asymmetry rather than enforcing symmetric gaps. However, existing studies remain heterogeneous and often underpowered, with conflicting results regarding the clinical impact of intraoperative balance on outcomes. Future research should rely on larger, prospective, high‐level evidence studies to better define optimal alignment–balancing strategies and their translation into functional benefit.

**Level of Evidence:**

Level V.

**Abbreviations**
BMI: body mass index
CPAK: Coronal Plane Alignment of the Knee
CT: computed tomography
FA: functional alignment
JLCA: joint line convergence angle
KA: kinematic alignment
MA: mechanical alignment
MRI: magnetic resonance imaging
OA: osteoarthritis
PS: posterior‐stabilized
PSI: patient‐specific instrumentation
TKA: total knee arthroplasty

## INTRODUCTION

Large registry and cohort studies consistently indicate that 15%–20% of total knee arthroplasty (TKA) patients remain dissatisfied at 1 year, with reported rates ranging from 5% to 40% [69, 83, 147]. Persistent pain, functional limitations, perceived instability and a mismatch between preoperative expectations and postoperative reality are the most commonly cited reasons [10, 65]. Beyond dissatisfaction, early revisions after TKA remain a major concern. Registry and multicenter data identify infection (24%–59%), instability (10%–26%) and stiffness (9%–18%) as the leading causes of revision within the first 2 years [60, 72, 106].

Among these, instability and stiffness could be directly linked to technical issues, particularly component malalignment and inadequate soft‐tissue balancing [110, 111]. These findings have challenged the long‐standing paradigm of strict mechanical alignment (MA). Alternative strategies such as kinematic alignment (KA), functional alignment (FA) and more recently the Coronal Plane Alignment of the Knee (CPAK) classification or functional knee phenotype (FKP) classification have been introduced to better capture inter‐individual anatomical variability [16, 36, 45, 48, 49, 51, 81].

In parallel, the concept of the physiological ‘laxity envelope’ has gained prominence as a determinant of functional knee stability. Biomechanical data demonstrate that the native knee is tighter in extension and looser in flexion, with marked medial‐lateral asymmetry [29, 112]. Accurate restoration of this envelope, rather than targeting a coronal alignment alone, could be essential to optimize postoperative kinematics and patient‐reported outcomes.

This dual perspective‐alignment versus ligament balancing remains at the core of ongoing debates. Alignment‐driven philosophies usually aim for predefined radiographic targets while balancing‐first approaches prioritize achieving an acceptable laxity profile, even at the expense of ‘ideal’ alignment. However, evidence suggests that neither alignment alone nor balance alone is sufficient to guarantee optimal outcomes.

The aim of this review is therefore to synthesize current knowledge on physiological knee laxity, alignment phenotypes and surgical philosophies in TKA, and to discuss how the interplay between alignment and ligament balancing may pave the way toward a personalized arthroplasty strategy.

### Descriptive concepts

#### Native and OA laxity patterns

The native knee exhibits a reproducible varus–valgus laxity envelope that increases with flexion. In full extension, coronal laxity remains low (approximately 2°–3°), with reported ranges between 0.5° and 5° for varus and 0.2°–4° for valgus, reflecting a near‐rectangular pattern with slightly greater lateral compliance [2, 34, 44].

**Figure 1:**
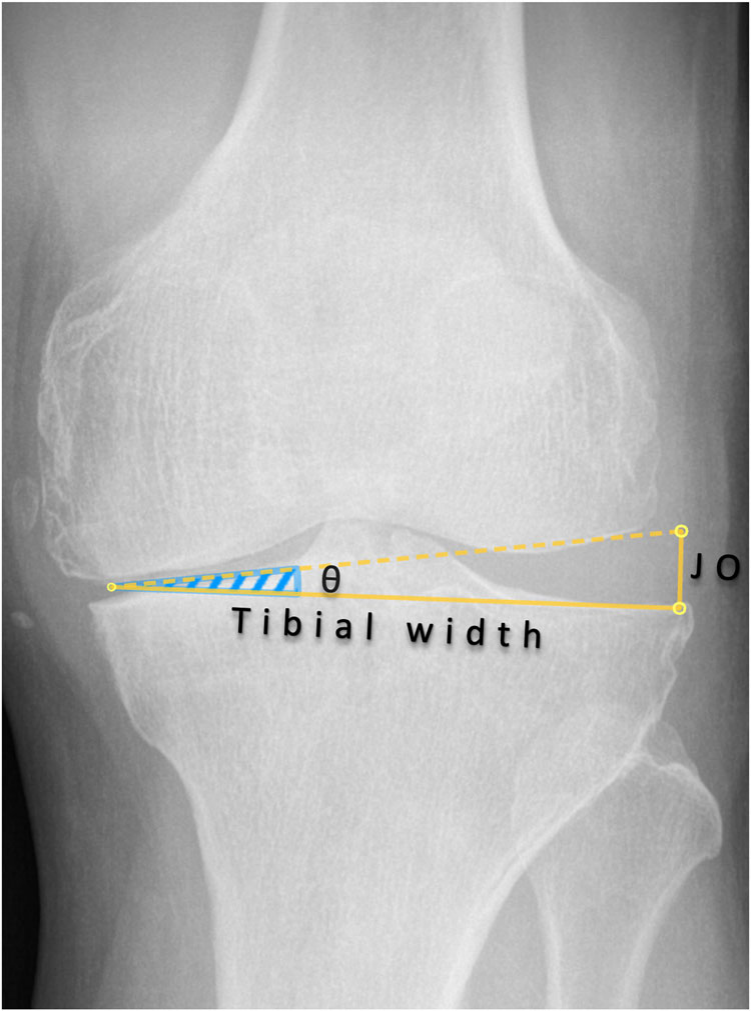
Relationship between angular laxity and joint opening in the coronal plane. Schematic illustration demonstrating the geometric relationship between angular varus–valgus laxity (*θ*, degrees) and medial–lateral joint opening (JO, mm) at the tibiofemoral level. For a given angular displacement, the resulting joint opening depends on the tibial width, according to the equation: Joint opening (mm) = tan(*θ*) × tibial width (mm). This relationship highlights that identical angular laxity values may correspond to different gap measurements depending on tibial morphology.

With increasing flexion, laxity progressively rises. At 30°–45°, varus laxity typically ranges from 3.5° to 4.5°, and at 90° it reaches approximately 4.5°–5°, with valgus values slightly lower [112, 131]. Magnetic resonance imaging (MRI)‐based stress analyses confirm greater lateral opening in flexion, with lateral gaps increasing by up to 7 mm under varus stress compared with approximately 2 mm medially under valgus stress [105]. Meta‐analytic data confirm these patterns while emphasizing the substantial inter‐individual variability in physiological laxity values [29]. Interpretation of physiological laxity values is limited by heterogeneity in measurement techniques, applied distraction loads, imaging modalities and study design (in vitro versus in vivo). In addition, some studies report laxity in degrees while others use millimetres, although these units are not interchangeable. Joint opening (mm) is mathematically related to angular laxity according to the relation:

Joint opening (mm) = tan(*θ*) × tibial width (mm) (Figure 1),

meaning that the same angular laxity may correspond to different gap values depending on tibial width.

Beyond methodological variability, substantial inter‐individual differences exist. Ethnic and population variations have been reported [7], and some studies describe greater laxity in women, although the independent effect of sex and body mass index (BMI) remains debated [27, 128]. Dynamic studies further demonstrate increased mediolateral laxity during gait, particularly in the presence of varus thrust [136], and this relationship is accentuated after repetitive stepping activity, with laxity rising by +0.05 m/s^2^ in controls versus +0.14 m/s^2^ in osteoarthritis (OA) patients. In summary, physiologically, the lateral compartment is consistently more lax, particularly in flexion, whereas the medial side acts as a stable anchor underpinning the concept of the medial pivot, albeit with significant inter‐ and intra‐individual variability [86, 148].

Coronal knee laxity in OA knees appears partly influenced by bony alignment and morphology. In a large robotic‐assisted TKA series (805 knees), medial ligament distractibility increased with the degree of varus deformity, whereas lateral distractibility slightly decreased, although substantial variability persisted within alignment groups [39]. OA alters the laxity envelope in a stage‐dependent manner. Early and moderate OA are associated with increased coronal and anteroposterior laxity [32], along with altered gait patterns OA [71, 76, 132]. In contrast, advanced medial OA may present smaller mediolateral differences under stress; Ishii et al. reported mean values of 4° medially and 3° laterally in Kellgren–Lawrence Grade 3–4 knees, with 90% remaining clinically balanced (≤ 3° difference in extension) [61]. Whether increased laxity in varus knees reflects reversible cartilage wear or structural alterations of the lateral ligament complex remains debated. In 240 varus OA knees, Colyn et al. demonstrated that progressive medial narrowing initially drives laxity, whereas lateral joint line opening exceeding 4.7 mm is associated with steep increases in coronal instability, suggesting irreversible ligamentous changes [19]. These findings align with gait analyses showing physiological variability of collateral laxity in healthy knees [22] and greater dynamic varus motion in OA patients with varus thrust, indicating ligamentous and neuromuscular involvement beyond cartilage loss alone [15]. Overall, despite heterogeneity across studies, OA is generally associated with increased laxity. However, most assessments remain static, whereas dynamic analyses suggest that instability may be accentuated during functional activity. Table 1 summarizes the key differences between native and OA knees regarding alignment, laxity patterns and their clinical implications.

**Table 1:** Key differences between native and OA knees in alignment, laxity patterns and clinical implications.

**Parameter**	**Native knee**	**OA knee**	**Key implications**
Coronal alignment (HKA) [9, 90, 108]	Mild constitutional varus common ( ≈ 25% >3° varus)	Varus drift predominates; mean ≈ −4° in TKA cohorts	OA progression amplifies individual constitutional alignment patterns, challenging the concept of a ‘standard’ OA deformity.
Joint line obliquity (JLO) [33, 67, 81]	Distal apex phenotypes frequent in healthy cohorts	Increased prevalence of varus–distal apex phenotypes in OA	Joint line obliquity is not uniform in OA, limiting the validity of standardized alignment targets.
Inter‐individual variability (alignment) [16, 46]	>120 functional phenotypes described	Subset observed in OA but still highly heterogeneous	No ‘average knee’ exists which supports personalization
Extension laxity (0°) [2, 44, 61].	Low coronal laxity ( ≈ 2°–3°); near‐rectangular	Often preserved; small ML difference in majority	Extension normally stable; over‐tightening risks stiffness
Flexion laxity (90°) [29, 39, 105]	Increased versus extension; lateral > medial	Increased and more variable medial distractibility in varus OA	Flexion more sensitive to pathology and balancing strategy
Medial compartment behaviour [19, 29]	Stable anchor (medial pivot concept)	Apparent medial opening possible in varus OA	Medial stability remains central to functional kinematics
Lateral compartment behaviour [15, 29, 105]	Greater compliance, especially in flexion	Dynamic instability in advanced OA (varus thrust)	Lateral laxity variability complicates balance targets
Sagittal alignment (PTS) [43, 89, 92]	Mean 5°–7°; wide range	Often increased; correlated with flexion deformity	PTS influences PCL tension and potentially flexion gap
Dynamic laxity (functional loading) [15, 22]	Physiologic variability under activity	Exaggerated dynamic varus motion in OA	Static measurements underestimate instability
Predictability of ligament behaviour from alignment [39, 74]	Weak correlation	Very weak correlation; high intra‐group variability	Bony alignment alone cannot predict soft‐tissue phenotype

Abbreviations: HKA, hip–knee–ankle; ML, machine learning; OA, osteoarthritis; PCL, posterior cruciate ligament; PTS, posterior tibial slope; TKA, total knee arthroplasty.

#### Coronal limb alignment in native and OA knees

Physiological alignment in healthy knees is not strictly neutral but tends toward a mild varus. Bellemans et al. showed in a large European cohort that nearly 25% of subjects displayed a constitutional varus higher than 3° [9]. This is consistent with computed tomography (CT)‐based analyses; Micicoi et al. reported a mean hip–knee–ankle (HKA) of 179.4° ± 2.6° [90], and Hirschmann et al. emphasized the wide variability of alignment phenotypes in non‐arthritic young adults. On average, the femur displayed a slight valgus and the tibia a slight varus, resulting in an overall alignment close to neutral or slightly varus [50].

Several studies have demonstrated sex‐ and age‐related variations. Men generally present with greater varus than women [9, 90]. Some authors have suggested constitutional bony remodelling related to age, characterized by an increase in lateral distal femoral angle (LDFA) and progressive coronal varus deformity, which may occur independently of any OA process [59]. There are also inter‐ethnic variations: importantly, Asian cohorts exhibit a stronger constitutional varus, averaging 2°–4°, with a higher prevalence of knees in varus >3°, but also a greater proportion of marked valgus deformities within the same population [42, 56, 90, 127]. In OA cohorts, these trends are accentuated, with varus phenotypes largely represented [94]. Similarly, Ramazanian et al. found a mean HKA of −4.1° with a wide standard deviation (± 6.1°) in patients awaiting TKA [108], reflecting both the varus shift and the increased variability induced by OA progression. These findings indicate that bony deformity and soft‐tissue behaviour should be considered together when planning TKA, particularly in the context of alignment personalization.

#### Sagittal parameters: tibial slope and its impact on kinematics and ligament balance

Posterior tibial slope (PTS) is a key sagittal parameter influencing knee biomechanics by modulating femoral rollback, tibiofemoral contact forces and cruciate ligament tension. In healthy populations, mean PTS ranges from 5° to 7°, with greater values typically observed in women [43], and wide inter‐individual variability reported (−5° to 25°) in cohorts undergoing TKA [89]. In OA knees, PTS tends to be increased and more variable, particularly in the presence of flexion contracture. Three‐dimensional weight‐bearing analyses of varus OA knees demonstrated significantly higher medial and lateral PTS in flexion contracture groups, with positive correlations between PTS and sagittal malalignment severity [92]. Although direct clinical evidence linking PTS to coronal laxity remains limited, biomechanical modelling suggests that increasing PTS reduces medial collateral ligament loading while slightly increasing forces in the lateral collateral and posterolateral structures, indicating that sagittal alignment may influence mediolateral stability [99].

### Classification systems

#### CPAK classification

While joint laxity reflects the dynamic soft‐tissue envelope of the knee, coronal alignment defines the static osseous framework within which this envelope operates. The CPAK classification [81], combining arithmetic HKA and joint line obliquity (JLO), illustrates the wide variability of alignment phenotypes. A systematic review including nearly 50,000 knees confirmed that neutral and mild varus phenotypes predominate in healthy populations, whereas varus phenotypes are more frequent in OA knees [33, 67, 70]. Geographic and sex‐related differences further emphasize the importance of contextualizing alignment within population norms. In European cohorts, neutral and mild varus phenotypes dominate, while in Asian series the distribution is skewed toward more extreme varus morphotypes [95, 107, 146]. Despite its pragmatic value, CPAK has notable limitations. Determination of the joint line apex may be inconsistent, with reported concordance below 50% [78, 114], and interobserver reliability ranges from poor to moderate in some studies [12]. Moreover, CPAK is restricted to coronal parameters and does not incorporate sagittal or rotational morphology [20, 93].

#### FKPs

Hirschmann's FKP classification [46, 50, 51, 94] describes coronal knee alignment using specific angular measurements of the femur, tibia and overall limb deformity. Each angle is stratified into categories (varus, neutral or valgus), and the combination of these categories defines an individual ‘phenotype′. For example, a knee may be classified as VARHKA3° + NEUFMA0° + NEUTMA0°, indicating the degree and direction of alignment for each segment; a global varus deformity of 3°, with NEUTMA0° and NEUFMA0° indicating respectively a 3° tibial varus deformity (TMA = 87°) and a 3° femoral valgus deformity (FMA = 93°). Considered neutral (NEU), since a 3° alignment represents the most frequent normative value observed in asymptomatic populations. This system highlights the wide variability of native alignment even in non‐OA populations and has been proposed to better capture individual differences and guide personalized surgical strategies. Large‐scale analyses have confirmed this variability, reporting 127 distinct phenotypes in men and 131 in women [16], owing to subtle differences in phenotypes. Not all theoretical phenotypes are observed in practice, and only a subset is commonly encountered in clinical populations. In a study of OA knees, for instance, 43 out of 125 possible phenotypes were identified [50, 51]. This classification also makes it possible to highlight inter‐individual differences depending on the patients' sex or geographic origin [16, 77]. In comparison to the CPAK, it has the advantage of being more comprehensive and offering more possible combinations to better define the individual phenotype of patients.

#### Laxity‐based classifications

**Figure 2:**
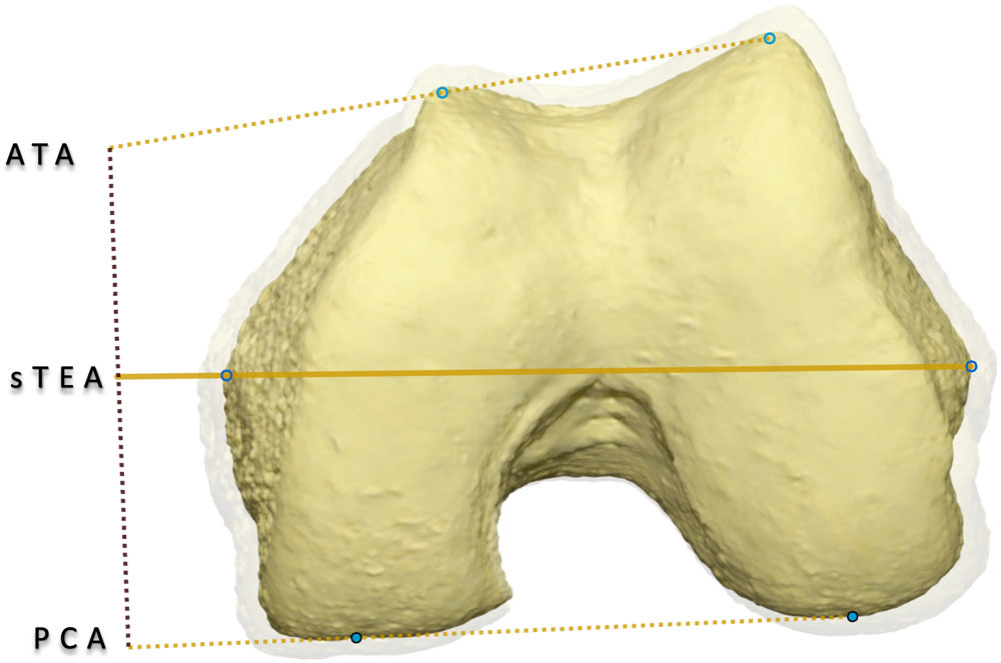
Three‐dimensional extension of the functional knee phenotype including rotational alignment. Three‐dimensional extension of the functional knee phenotype illustrating the posterior condylar angle (PCA) and anterior trochlear angle (ATA) relative to the surgical transepicondylar axis (sTEA). These parameters account for rotational and trochlear variability not captured by coronal alignment alone.

Graichen and Hirschmann introduced the concept of laxity‐FKPs by simulating gap patterns under different alignment workflows. MA consistently produced lateral laxity in flexion (98%), whereas patient‐specific resections yielded a broader distribution but still with marked intra‐phenotype variability. When patient‐specific bony restorations (B‐FKP) were applied, the resulting laxity phenotypes in flexion were highly variable, with just over half of knees (56%) showing lateral laxity, nearly one‐third (31%) demonstrating neutral balance, and a smaller proportion (13%) presenting medial laxity. Importantly, correlations between coronal alignment and laxity patterns—in flexion and in extension—were weak, underscoring again that deformity does not dictate ligament phenotype [37]. In a complementary analysis, Graichen et al. introduced the SIPR (Scoring for Individual Phenotype Restoration), a three‐dimensional framework that integrates bone resections, gap balance and alignment parameters to quantify how different workflows restore both bony and ligamentous phenotypes. Using a simulator across varus, valgus and neutral CPAK phenotypes, they showed that while neutral knees scored similarly across all strategies, personalized workflows outperformed MA in varus and valgus morphotypes [38]. Completing this three‐dimensional framework, Hirschmann's group proposed an extension of the FKP concept to include rotational alignment parameters, namely, the posterior condylar angle (PCA) and anterior trochlear angle (ATA) (Figure 2), in addition to the distal femoral and proximal tibial joint lines [45]. In a large cohort of more than 2900 knees, they demonstrated that coronal and rotational phenotypes are largely independent, with only 2%–3% of individuals showing congruence across all four joint lines. This reinforces that patients sharing the same coronal phenotype may still display highly variable femoral rotation, underscoring the importance of incorporating anterior and posterior femoral joint lines into preoperative planning and potentially moving toward more individualized alignment and implant designs [45].

Recent research has emphasized that coronal bony alignment alone does not explain the variability in intraoperative ligament balance during TKA. Grosso et al. analysed 805 varus knees undergoing robotic‐assisted TKA and demonstrated that medial ligament distractibility increased with varus severity, while lateral distractibility slightly decreased. However, substantial variability existed within each alignment category (HKA, medial proximal tibial angle, LDFA), with standard deviations of 2–3 mm, indicating that identical varus deformities may correspond to markedly different laxity profiles. The authors concluded that although deformity influences gap patterns, bone alignment alone cannot reliably predict individual soft‐tissue balance [39]. Similarly, Kim et al. evaluated 1124 robotic TKAs using digital tensioning and found that varus CPAK phenotypes tended to exhibit larger medial gaps, whereas valgus knees showed inconsistent patterns. Despite observable trends, high intra‐phenotype variability (standard deviation [SD] up to ±5.5 mm) limited predictive value. Differences from prior reports were likely related to cartilage wear correction applied to CPAK classification but not to intraoperative gap measurements. The authors concluded that although CPAK trends exist, bone morphology alone cannot predict ligament balance, reinforcing the need for intraoperative tensioning [66]. The observation of larger medial gaps in varus CPAK phenotypes, contrary to previous reports [101, 112], could be explained by the methodological distinction that cartilage wear correction was applied when calculating bony alignment for CPAK classification, whereas intraoperative gap measurements remained uncorrected and therefore reflected the arthritic joint space. In a larger cohort of 4362 robotic TKAs, Lee et al. confirmed that while varus phenotypes generally showed greater medial laxity and valgus phenotypes greater lateral laxity, considerable overlap existed between CPAK classes. Even after simulating cartilage restoration, substantial intra‐phenotype variability persisted. Although adjusting CPAK thresholds improved discrimination, it did not resolve the variability in ligament balance. The authors concluded that CPAK captures common bony morphologies but fails to reflect the individuality of the soft‐tissue envelope, limiting its predictive value for intraoperative balance [74].

Kenanidis et al. recently introduced the REAL (Robotic Evaluation of Articular Laxity) classification, which categorizes soft‐tissue balance intraoperatively into nine phenotypes based on mediolateral differences in extension (1,2,3) and flexion (A,B,C) leading to distinct phenotypes defined by thresholds of 2.5 mm of imbalance (for example, 1C indicates <2.5 mm difference in extension and >5 mm in flexion). This system, validated in over 200 robotic TKAs, could simplify the description of ligament balance by identifying a reproducible ‘balanced zone′ (Classes 1A/1B) achieved in 93% of cases according to the authors [64].

In summary, across these studies, coronal bony deformity shows limited predictive value for intraoperative ligament behaviour. Novel concepts such as L‐FKP and REAL classification highlighted that each knee possesses a unique ‘ligament phenotype’ that may diverge substantially from its bony alignment phenotype. At present, no consensus exists on how best to integrate these classifications into surgical decision‐making, and further research is required to define reproducible balancing targets.

### Surgical alignment philosophies

#### Alignment philosophies

MA has been the dominant TKA philosophy since the 1970s, aiming to implant femoral and tibial components perpendicular to their mechanical axes to achieve a neutral hip–knee–ankle angle and a horizontal joint line. This approach provides even load distribution and excellent long‐term survivorship, as demonstrated in large cohorts showing low revision rates and comparable functional outcomes between neutrally aligned and outlier knees at long‐term follow‐up [1, 28, 109]. More personalized strategies subsequently emerged. Anatomical alignment (AA), introduced by Hungerford and Krackow [57], targeted a slightly oblique joint line (2°–3°) to better reproduce physiological anatomy. Although early reports raised concerns regarding polyethylene wear, recent randomized studies using robotic assistance have shown no significant differences in outcomes or implant survival between AA and MA [97, 144]. KA, described by Howell in 2008, represented a conceptual shift toward resurfacing the knee according to the patient's pre‐arthritic anatomy, aiming to preserve the native joint line and minimize ligament releases [52, 54, 75]. While some randomized trials reported improved early functional scores with KA compared with MA [23], others demonstrated equivalent mid‐term outcomes [137, 145]. To mitigate concerns regarding extreme alignment, derivative approaches such as restricted KA, inverse KA and FA were developed. Enabled by navigation and robotics, these techniques constrain alignment within predefined boundaries or dynamically adapt component positioning based on intraoperative soft‐tissue balance [3, 58, 62, 125, 126, 134]. Collectively, they are now encompassed under the concept of ‘personalized alignment’, reflecting a shift away from uniform mechanical targets toward individualized reconstruction [47, 79].

#### Measured resection versus gap‐balancing

Overall, two opposing approaches can be distinguished in the literature: measured resection and gap‐balancing. In the measured resection technique, bone cuts are planned to match implant thickness, using anatomical landmarks such as the transepicondylar axis, posterior condylar axis or Whiteside's line. The goal is to restore the native joint line with minimal need for soft‐tissue release. This principle is central to KA, where caliper‐verified resections resurface the joint in a patient‐specific manner [53, 118]. By contrast, the gap‐balancing technique focuses more on the ligaments, prioritizes achieving symmetric and rectangular extension and flexion gaps and often adjusts femoral component rotation or tibial resection to equalize ligament tension. This approach is embedded in modern FA, facilitated by navigation or robotic platforms that provide intraoperative feedback on bone resections and soft‐tissue balance [21, 96].

Moreover, alignment philosophy strongly influences trochlear anatomy restoration, particularly in valgus knees (LDFA < 84°); kinematic, restricted kinematic (rKA) and FA best reproduce native trochlear orientation and sulcus position, whereas mechanical and gap‐balancing approaches caused up to 9° of deviation and greater trochlear understuffing [103]. These findings suggest that individualized alignment also plays a role in better restoring patellofemoral congruence and improving tracking compared with traditional MA. Understanding the distinction between these approaches is critical, as they frame the evolution from uniform, mechanically oriented TKAs toward more personalized strategies.

#### Comparative clinical results

The clinical evidence comparing alignment philosophies demonstrates that personalized approaches achieve at least equivalent, and in some cases superior, outcomes to MA. Dossett et al. showed that patients treated with KA had significantly better Western Ontario and McMaster Universities Arthritis Index (WOMAC), Oxford and Knee Society Scores (KSSs) at 2 years compared with those treated with MA, with improved flexion as well [23]. Similarly, Calliess et al. found higher combined KSS and WOMAC scores for KA at 1 year using patient‐specific instrumentation [14].

In contrast, Waterson et al. and Young et al. reported no statistically significant differences in patient‐reported outcome measures (PROMs), range of motion or implant migration between KA and MA at mid‐term follow‐up, suggesting that the superiority of KA is not universally reproducible [137, 145]. Long‐term studies remain scarce and have to be analysed, but some have reported excellent outcomes, such as Howell et al., who documented survivorship exceeding 97% at 10 years for unrestricted KA, comparable with traditional MA [55].

Schelker et al. [116] performed simulation analyses of four alignment strategies: MA, AA, KA and rKA across five common varus phenotypes identified by the Hirschmann classification. They showed that in knees with mild deformity (near‐neutral phenotypes), the choice of alignment had little effect on joint line height or obliquity. In contrast, in severe varus phenotypes, particularly those with oblique joint lines, imposing MA or AA required large asymmetric resections, potentially leading to ligament imbalance and the need for extensive release. KA preserved the native joint line, while rKA offered a compromise by limiting deviations into a ‘safe zone’. The authors concluded that the optimal alignment strategy should be individualized to the knee phenotype rather than applied dogmatically.

Tachibana et al. [130] compared MA and modified kinematic alignment (mKA) stratified by CPAK type. In CPAK Type I knees, mKA resulted in slightly better outcomes than MA: mean postoperative flexion was 124° versus 118°, lateral laxity in flexion was better preserved (3.0 vs. 1.5 mm) and patient satisfaction rates were higher (92% vs. 76%). In CPAK Type II knees, these advantages disappeared; postoperative flexion averaged 119° with no clear superiority of mKA compared with MA. These findings suggest that the clinical benefit of mKA could be phenotype‐dependent and that alignment techniques may have little influence on certain phenotypes, such as CPAK Type II, which remains largely represented among OA patients.

Ettinger et al. [85] demonstrated that rKA provided significantly better functional outcomes than MA in varus CPAK knees, with higher Forgotten Joint Scores (63.1 vs. 44.9 at 1 year, *p* = 0.036; 71.1 vs. 46.0 at 2 years, *p* = 0.005), as well as superior WOMAC and KSS satisfaction scores. In contrast, in neutral CPAK types, no significant differences were observed between rKA and MA. Beyond CPAK, the authors also reported that for Hirschmann phenotypes: under MA, knees frequently shifted into altered phenotypes with more asymmetric joint lines, whereas rKA tended to preserve native phenotypes such as VARFMA3°/NEUTMA0°, highlighting its potential to maintain constitutional morphology.

Franceschetti et al. [31] evaluated outcomes of mechanically aligned TKA and also found that results were not uniform across CPAK subtypes. Patients with varus aHKA phenotypes had significantly worse functional and satisfaction scores (KSS 79.7 vs. 85.6; Oxford Knee Score [OKS] 39.2 vs. 42.2; Forgotten Joint Score [FJS] 75.4 vs. 87.4) compared with other categories, whereas neutral and valgus types showed no significant differences. Similarly, Pangaud et al. reported a slight improvement in functional outcomes when the constitutional aHKA was restored postoperatively [140].

Overall, personalized alignment strategies, including KA, rKA, iKA and FA, appear to reduce the need for collateral ligament releases and may better respect native joint anatomy [91]. However, systematic reviews emphasize that differences between techniques are small, complication rates are similar and definitive long‐term data are lacking [119]. High‐quality randomized trials with extended follow‐up will be essential to determine whether personalization of alignment translates into durable improvements in survivorship and in function, particularly whether these functional gains exceed the minimal clinically important difference (MCID).

#### Objective intraoperative assessment of ligament balance

The various robotic and navigation systems assess varus–valgus laxity using heterogeneous metrics (millimetres, degrees or applied load), while bone resections, implant sizing and surgeon‐applied forces during acquisition can further influence measured laxity and are not fully reproducible. In the absence of standardized references, ligament balancing therefore remains largely surgeon‐dependent and experience‐based, limiting comparisons across studies. Accurate intraoperative measurement of medial and lateral gaps is essential for optimal soft‐tissue balance in TKA, yet it is highly sensitive to the applied distraction force. Increasing the load from 60 to 80 N results in larger measured gaps and stronger correlations with mediolateral laxity, particularly at mid‐flexion [141]. Similarly, distraction forces increased from 70 to 90 N have been shown to widen flexion gaps by approximately 1–3.2 mm, predominantly on the lateral side [123]. Using a navigated seesaw digital balancer applying standardized, non‐manual forces, Seito et al. demonstrated in 55 posterior‐stabilized (PS) TKAs that although both medial and lateral gaps increased with force, the medial extension–flexion gap difference remained constant from 20 to 60 lb, making it a reliable intraoperative reference. In contrast, lateral gaps and varus angles varied more with force, reflecting greater lateral laxity in flexion and supporting the use of the medial gap to guide femoral rotation and posterior condylar resection in PS‐TKA [120]. Robotic and instrumented tensioners enable controlled, quantifiable distraction forces and provide more consistent and reproducible gap measurements throughout the range of motion than manual techniques, which are subject to surgeon‐dependent variability [13, 122, 123]. This was further confirmed in a cadaveric navigated TKA study by Boux de Casson et al., who showed that force‐controlled ligament tensioning devices achieved significantly higher reproducibility of medial and lateral gap measurements than manual varus–valgus stress testing, regardless of surgeon experience [13].

This inter‐ and intra‐individual variation in applied force can lead to under‐ or over‐tensioning after TKA, potentially affecting joint stability or stiffness. When comparing empirically applied varus–valgus forces by five surgeons during intraoperative testing, differences of up to 2 mm in measured laxity were observed, highlighting substantial inter‐individual variability in soft‐tissue balance [25]. Hand‐held gap spreaders have similarly been shown to overestimate laxity and create asymmetries, particularly in flexion and on the lateral side, supporting the use of devices that standardize or quantify applied force (tensioners or sensors) for more reliable ligament balancing during TKA [30]. In robotic‐assisted TKA, Sohmiya et al. demonstrated that manual varus/valgus stress using a Z‐retractor is highly operator‐dependent and tends to underestimate joint laxity, especially in flexion [129]. Conversely, other studies have reported that robotic‐assisted workflows can provide objective, real‐time assessment of ligament balance during manual varus–valgus stress, with good‐to‐excellent inter‐ and intra‐rater reliability for pre‐resection gap measurements in all compartments except lateral flexion [117, 143]. However, when robotic systems rely on preoperative CT‐based laxity predictions, real‐time intraoperative adjustments remain necessary, as medial–lateral gap differences may change by up to ±1 mm in extension and ±2 mm in flexion after bone cuts [84]. Woelfle et al. prospectively compared manual varus/valgus stress without force calibration and a ligament‐tensor device applying a standardized 90 N per compartment during robotic‐assisted TKA in 50 knees. Both methods produced nearly identical gap measurements and implant planning parameters, with only minor, non‐significant differences, although the manual approach was faster [138].

Elmallah et al. questioned whether surgeons can reliably ‘feel’ a balanced knee and showed that intraoperative sensor technology provides a more objective and reproducible assessment of compartmental loads [24]. This need for objective intraoperative data has driven the adoption of quantitative sensor systems, such as Verasense, either alone or combined with robotic workflows. Sensor‐guided TKA achieves a higher rate of quantitatively balanced knees compared with manual techniques, with more than 90% balanced within defined pressure thresholds, versus 35%–37% after initial bone cuts [18, 26, 80, 113]. Despite this, distraction forces used to achieve ligament balance remain highly heterogeneous. A recent meta‐analysis reported mean forces of approximately 150 N in extension (range: 35.0–320.0 N) and 140 N in flexion (range: 14.7–244.7 N), which may serve as reference benchmarks for future studies on intraoperative soft‐tissue balancing [8].

### Clinical implications of surgical alignment philosophies in TKA

#### Alignment philosophy and ligament balancing interaction

The interaction between alignment strategy and soft‐tissue balance is central to achieving functional knee kinematics, as alignment choices influence both bone resections and ligament tension patterns. In a randomized trial of 116 TKAs, Lee et al. showed that navigation‐assisted gap balancing resulted in fewer soft‐tissue balancing outliers (12% vs. 25%) and fewer medial flexion–extension gap asymmetries (5% vs. 23%) than conventional measured resection, despite similar short‐term clinical scores [73]. Similarly, in 100 robotic‐assisted TKAs using a tibia‐first restricted FA approach, Yang et al. demonstrated that balanced gaps (≤2 mm differential) were achieved in 98% of cases, even though bone resections produced variable medial (up to 1.6–1.7 mm) and lateral (≈1.0 mm) gap changes. The tibial component was positioned around 2° varus and the femoral component near neutral with slight external rotation, with significant improvement in 1‐year PROMs, supporting the reproducibility of intraoperative balance with this technique [142].

In a randomized cohort of 138 TKAs, KA achieved objective soft‐tissue balance (mediolateral load differential ≤15 lb in extension and 90° flexion) more frequently than MA, particularly in CPAK Types I, II and IV (100% vs. 15% balanced in Type I), suggesting that tailoring alignment strategy to CPAK phenotype improves intraoperative balance [5, 81]. However, analysis of over 4000 robotic TKAs showed substantial overlap in extension gap balance across all CPAK phenotypes, indicating that osseous alignment alone cannot reliably predict ligament behaviour and underscoring the need to account for the individual soft‐tissue envelope [74]. Similarly, in a randomized bilateral trial, McEwen et al. reported comparable functional outcomes but fewer soft‐tissue releases and greater patient preference with KA, likely related to restoration of a more anatomic joint line rather than neutral alignment [87]. In a series of 102 varus knees undergoing robotic‐assisted TKA, Shatrov et al. showed that a caliper‐based KA plan achieved mediolateral balance in only 65.7% of extension gaps and 49.1% of flexion gaps, whereas subsequent adjustments toward FA achieved balance in nearly all cases with minimal soft‐tissue releases. These findings indicate that an individualized FA approach, incorporating intraoperative ligament tension measurements, more reliably achieves balance than caliper‐based KA alone in varus knees [124].

In a robotic study of 141 TKAs, both rKA and gap balancing reproduced native medial stability with lateral flexion laxity. Gap balancing achieved slightly more symmetrical gaps but with 1–2 mm greater overall laxity, whereas rKA produced a tighter construct with increased joint‐line obliquity and a higher rate of tibial recuts (26.5% vs. 1.4%) [104]. Despite patient‐specific design, customized implants do not integrate real‐time ligament balancing, leaving soft‐tissue adjustment dependent on intraoperative judgement; accordingly, tibial recuts were required in 46% of 258 custom TKAs in Bonnin et al.'s series [11]. Although robotic systems and predictive tensioning devices improve intraoperative gap symmetry, randomized trials have not demonstrated clinically meaningful differences in patient‐reported outcomes at 2 years compared with manual techniques, with higher rates of ‘balanced’ knees failing to consistently exceed MCID or Patient Acceptable Symptom State (PASS) thresholds [63, 135]. Finally, posterior osteophytes smaller than 10 mm have minimal impact on coronal balance, with mean gap changes of ~1 mm after removal, suggesting they can be ignored during initial alignment and gap optimization [40].

#### Postoperative laxity and its functional impact: Searching for the optimal balance window

Golladay et al. showed that quantitatively balanced TKAs (mediolateral load differential ≤15 lb) were associated with higher Forgotten Joint and Knee Society Satisfaction scores at 6 weeks and 6 months compared with unbalanced knees, supporting the benefit of intraoperative sensor‐guided balance on early PROMs [35]. However, randomized trials and systematic reviews have not demonstrated significant differences in PROMs, range of motion or reoperation rates at 1–2 years between sensor‐guided and manual balancing [82, 115, 139]. Similarly, Meneghini et al. found that patients with either a rectangular flexion gap or mild lateral laxity after PCL resection reported less pain and higher satisfaction at a mean 1.5‐year follow‐up than those with medial laxity, supporting the functional benefit of preserved posterolateral rollback and helping define clinically relevant flexion‐gap targets [88].

Valpiana et al. compared two KA techniques using medially stabilized implants: an ‘unrestricted’ KA with symmetric gaps and a robot‐assisted ‘simplified’ KA with intentionally tighter medial gaps (1.5 mm in flexion, 2 mm in extension compared to lateral gaps). At 9 months, gait analysis showed that the asymmetric group (medial tight, lateral loose) achieved significantly greater knee flexion during walking and a motion pattern closer to the native knee, despite similar coronal alignment. These findings suggest that reproducing mild medial tightness and lateral laxity may better restore physiological kinematics after TKA [133]. Consistent with these observations, Nakamura et al. analysed 656 TKAs using a standardized 178 N tensioning device and found that medial laxity exceeding 5 mm was associated with inferior postoperative function and satisfaction, both in extension and flexion, with the strongest negative correlation observed in flexion. In contrast, comparable lateral laxity did not adversely affect outcomes, reinforcing the concept of an optimal balance window characterized by medial stability and controlled lateral laxity [98].

More recently, in a large series of over 500 cruciate‐retaining TKAs, it was shown that lateral flexion laxity up to 6 mm does not compromise clinical outcomes; patients with moderate laxity between 3 and 6 mm achieved higher functional and satisfaction scores and reported greater ease in stair descent, suggesting that controlled lateral looseness in flexion may be beneficial when medial stability is preserved [100]. Hamilton et al. evaluated 42 well‐functioning TKAs to determine whether residual ligamentous laxity influenced outcomes. Despite wide variability in coronal and sagittal laxity among asymptomatic patients, no significant differences were found in range of motion, Oxford Knee Score, functional tests or satisfaction. These findings suggest that a certain degree of postoperative laxity may be well tolerated and that an individualized ‘balance window’ rather than a universally tight construct may better reflect functional stability after TKA [41].

Ideally, reproducing the native ligament balance is key to restoring normal joint perception after surgery; however, accurately determining the pre‐OA soft‐tissue condition remains challenging due to the structural changes induced by OA progression. While achieving a well‐balanced knee seems essential, it is equally important to avoid excessive tightness in extension; Okamoto et al. showed that maintaining more than 1 mm of laxity in the extension gap is necessary to prevent postoperative flexion contracture [102].

#### Defining the postoperative target and future research perspectives

The absence of clinically meaningful differences in some studies may partly reflect the lack of accounting for muscular activation intraoperatively. Experimental data show that postoperative laxity changes under active loading, as quadriceps and hamstring co‐contraction alter joint stability compared with passive conditions, suggesting that intraoperative laxity alone may not fully reflect functional stability [6, 121]. In a robotic TKA cohort, Andriollo et al. introduced coronal alignment at 90° of flexion (rHKA‐90F) as a dynamic parameter, showing that patients maintaining approximately 5° of varus in flexion achieved higher functional and satisfaction scores, supporting the benefit of mild varus alignment and controlled lateral laxity in flexion [4]. Klasan et al. further emphasized that FA represents an umbrella concept rather than a single technique; in robotic simulations, FA was more often achieved by preserving tibial alignment and allowing up to 6 mm of controlled lateral flexion laxity. They also highlighted the need for standardized nomenclature defining the starting alignment, preserved component and targeted gap symmetry to improve comparability and guide future research [68].

Analysing nearly 3000 knees, Hess et al. found that only 2%–17% showed parallel alignment across femoral and tibial planes, with weak correlations between coronal and rotational parameters (*r* < 0.3) and wide variability in posterior (±5°) and anterior trochlear (±10°) femoral angles. These findings support a three‐compartment phenotype concept integrating femorotibial rotation and patellofemoral morphology to better capture three‐dimensional alignment variability and guide implant personalization [45]. Beyond alignment, implant conformity, PTS and tibial tray rotation also influence ligament balance, as higher conformity and excessive slope or malrotation increase sensitivity to malalignment, underscoring the need for optimized component design to preserve physiological laxity [17].

## CONCLUSION

TKA is evolving from a one‐size‐fits‐all mechanical procedure to a more patient‐specific restoration of each knee's unique anatomy and ligament behaviour. The growing body of evidence demonstrates that neither alignment nor ligament balance alone guarantees success; it is their interplay that defines functional stability and patient satisfaction. Understanding each knee's bony and soft‐tissue phenotype allows surgeons to better reproduce the native medial pivot and controlled lateral laxity that underlie physiological kinematics. Modern tools, including sensors, robotics and quantitative tensioning devices, make it possible to precisely measure rather than estimate this balance window. The future of TKA lies in dynamic, data‐driven personalization, where alignment, laxity and morphology converge to restore not only motion but also the patient′s own ‘feeling of normality’.

## AUTHOR CONTRIBUTIONS

**Grégoire Micicoi** and **Sébastien Lustig:** Manuscript writing. **Peter K. Sculco, Stefano Zaffagnini, Friedrich Boettner** and **Michael T. Hirschmann:** Article review. All authors approved the final manuscript.

## CONFLICT OF INTEREST STATEMENT

The authors declare no conflict of interest.

## ETHICS STATEMENT

The authors have nothing to report.

## Data Availability

The authors have nothing to report.
